# Attenuation of Inflammatory Responses in Breast and Ovarian Cancer Cells by a Novel Chalcone Derivative and Its Increased Potency by Curcumin

**DOI:** 10.1155/2023/5156320

**Published:** 2023-01-12

**Authors:** Mitra Nourbakhsh, Shokoofe Noori, Zahra Aminzade, Maryam Bayanati, Mehrdad Alemi, Afshin Zarghi

**Affiliations:** ^1^Finetech in Medicine Research Center, Iran University of Medical Sciences, Tehran, Iran; ^2^Department of Biochemistry, School of Medicine, Iran University of Medical Sciences, Tehran, Iran; ^3^Department of Biochemistry, Faculty of Medicine, Shahid Beheshti University of Medical Sciences, Tehran, Iran; ^4^School of Pharmacy, Shahid Beheshti University of Medical Sciences, Tehran, Iran

## Abstract

**Background:**

Breast and ovarian cancers are two common malignancies in women and a leading cause of death globally. The aim of the present study was to explore the effects of a novel chalcone derivative 1-(4-(methylsulfonyl)phenyl)-3-(phenylthio)-3-(p-tolyl)propane-1-one (MPP) individually or combined with curcumin, a well-known herbal medicine with anticancer properties, as a new combination therapy on inflammatory pathways in breast and ovarian cancer cell lines.

**Methods:**

LPS-induced NF-*κ*B DNA-binding activity and the levels of proinflammatory cytokines were measured in the MPP- and MPP-curcumin combination-treated MDA-MB-231 and SKOV3 cells by ELISA-based methods. The expression of *COX2*, *INOS*, and *MMP9* genes and nitrite levels was also evaluated by real-time qRT-PCR and Griess method, respectively. I*κ*B levels were evaluated by Western blotting.

**Results:**

MPP significantly inhibited the DNA-binding activity of NF-*κ*B in each cell line and subsequently suppressed the expression of downstream genes including *COX2*, *MMP9*, and *INOS*. The levels of proinflammatory cytokines, as well as NO, were also decreased in response to MPP. All the effects of MPP were enhanced by the addition of curcumin. MPP, especially when combined with curcumin, caused a remarkable increase in the concentration of I*κ*B.

**Conclusion:**

MPP and its coadministration with curcumin effectively reduced the activity of the NF-*κ*B signaling pathway, leading to a reduced inflammatory response in the environment of cancer cells. Thus, MPP, either alone or combined with curcumin, might be considered an effective remedy for the suppression of inflammatory processes in breast and ovarian cancer cells.

## 1. Introduction

Cancer is one of the significant health problems around the world. Breast cancer is the most frequent cancer and the global leading cause of cancer mortality in women [1]. Among the different types of breast cancer, the triple negative is the most invasive type of breast cancer and the hardest to treat [2]. Ovarian cancer is far less common but is the most lethal gynecological malignancy due to the late diagnosis of more than 70% of cases [3, 4]. Various therapeutic strategies, including surgery, radiotherapy, immunotherapy, hormone therapy, and chemotherapy, with different therapeutic mechanisms and targets, are used for the treatment of these cancers [5, 6]. However, an important challenge that still lies ahead is that most of these chemotherapeutic agents do not fulfil the desirable outcome. Therefore, the urgent need to develop novel anticancer drugs has led scientists to explore the world of medicinal plants and synthesize new antineoplastic compounds.

An active area in cancer research is the use of natural products and their derivatives in the prevention and treatment of cancer [7]. Chalcones are aromatic-containing unsaturated ketones with various substituents that are the precursors of flavonoids [8]. These compounds, either naturally occurring or synthetic, possess a wide variety of biological properties, including activity against cancer growth and metastasis [9]. Curcumin, a hydrophobic polyphenol extracted from the rhizomes of turmeric (*Curcuma longa* L.), is extensively studied for its various pharmacological effects, including anticancer properties [10, 11]. Curcumin has shown promising results in treating cancer, both alone and in combination with other chemotherapeutic agents. It exerts anticancer effects, mainly by modulating various signaling pathways and transcription factors [12, 13].

One of the hallmarks of cancer is the activation of nuclear factor-*κ*B (NF-*κ*B), a transcription factor crucial for inflammatory responses, which links chronic inflammation to cancer. Activation of NF-*κ*B leads to the production of proinflammatory, proliferative, and antiapoptotic agents and results in the promotion of invasion, angiogenesis, and the suppression of antitumor immunity [14]. The main event in the canonical pathway for the activation of NF-*κ*B is the phosphorylation of its inhibitor, I*κ*B, by I*κ*B kinase (IKK), which leads to its degradation and separation from NF-*κ*B. The release of NF-*κ*B enables its translocation into the nucleus, resulting in the transcription of the NF-*κ*B target genes [15]. NF-*κ*B activation leads to the upregulation of inducible nitric oxide synthase (iNOS). iNOS catalyzes the formation of nitric oxide (NO), an essential cellular signaling molecule involved in inflammatory processes and angiogenesis [16]. NF-*κ*B induces the production of proinflammatory cytokines such as TNF-*α*, IL-1*β*, and IL-6 and upregulates proinflammatory enzymes like cyclooxygenase-2 (COX2) [17–19]. Inflammation is related to all the stages of cancer development and progression, as well as to the effectiveness of anticancer treatments. Chronic inflammation results in immunosuppression; thus, it provides a microenvironment that favors tumorigenesis and metastasis [20]. NF-*κ*B can also contribute to tumor invasion by induction of matrix metalloproteinase 9 (MMP9) that degrades the extracellular matrix and provides a suitable niche for the invasion of cancerous cells [21].

Given the pivotal role of the NF-*κ*B signaling pathway in carcinogenesis, one of the promising pharmacological strategies in the treatment and prevention of cancer is the suppression of NF-*κ*B pathway. There has been much effort in recent years to explore drugs that block NF-*κ*B activity [22]. In the current study, we investigated the effect of a novel chalcone derivative with the chemical name 1-(4-(methylsulfonyl)phenyl)-3-(phenylthio)-3-(p-tolyl)propane-1-one abbreviated as MPP, alone or combined with curcumin, on the NF-*κ*B signaling pathway, in two different cancer cell lines including MDA-MB-231 triple-negative breast cancer cell line and the SKOV3 ovarian cancer cell line.

## 2. Materials and Methods

### 2.1. Synthesis of 1-(4-(Methylsulfonyl)phenyl)-3-(phenylthio)-3-(p-tolyl)propan-1-one (MPP)

**Figure 1:**
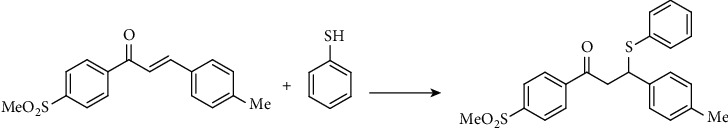
The reaction of the synthesis of 1-(4-(methylsulfonyl)phenyl)-3-(phenylthio)-3-(p-tolyl)propan-1-one (MPP).

Thiol (3 mmol) was added to a mixture of chalcone (2.0 mmol) in chloroform (4 mL), while stirring until the color changed from yellow to white and TLC showed completion of the reaction. After evaporation of volatiles, the residue was washed with hexane and crystallized using methanol (yield: 91%). The reaction and chemical structure of the produced compound named MPP are presented in Figure 1.

Chemical and physical properties are as follows: white powder; mp: 118–120°C; IR (KBr): *ν* (cm^−1^) 1157, 1325 (SO_2_), 1679 (C=O); ^1^H NMR (CDCl_3_): 2.28 (s, 3H, Me), 3.05 (s, 3H, SO_2_Me), 3.54-3.68 (m, 2H, C*H_2_*), 4.89 (t, 1H, C*H*), 7.07 (d, 2H, 4-methylphenyl *H*_2_ and *H*_6_, *J* = 8 Hz), 7.21-7.34 (m, 7H, 4-methyl phenyl *H*_3_ and *H*_5_ and phenylthio), 8.01 (s, 4H, 4-methylsulfonylphenyl); ^13^C NMR (CDCl_3_): 21.09, 44.26, 45.31, 47.92, 127.55, 127.66, 127.76, 128.89, 128.94, 129.27, 132.69, 134.10, 137.32, 137.56, 140.61, 144.18, 196.12; LC-MS (ESI) *m/z*: 411 (M+1). Anal. Calcd. for C_23_H_22_O_3_S_2_: C, 67.29; H, 5.40. Found: C, 67.52; H, 5.66.

### 2.2. Cell Culture

The human MDA-MB-231 breast cancer cell line and SKOV3 ovarian cancer cell line were obtained from Pasteur Institute of Iran. MDA-MB-231 cells were grown in DMEM, supplemented with 10% fetal bovine serum (FBS), penicillin (100 U mL^−1^), and streptomycin (100 *μ*g mL^−1^). The SKOV3 ovarian cancer cells were cultured in RPMI-1640 medium containing 10% FBS and 1% penicillin/streptomycin. Both cells were maintained at 37°C in a humidified atmosphere with 5% CO_2_/95% air.

### 2.3. Cell Viability Assay

Cells were seeded (1 × 10^4^ cells/well) in 96-well plates and incubated at 37°C with 5% CO_2_. After 24 h, the cells were treated with LPS (1 *μ*g/mL in 100 *μ*L medium) to stimulate an inflammatory response. A group of cells were pretreated with 5 to 100 *μ*M concentrations of MPP for 24 h, prior to stimulation with LPS. Another group was pretreated with a combination of MPP and curcumin (10 *μ*M) (Sigma-Aldrich, Germany), followed by treatment with LPS. Curcumin was solubilized in 0.5 M NaOH and then immediately diluted in phosphate-buffered saline (PBS). Treated cells were incubated for 24 h. MTT assay was performed to assess the inhibitory effect of each treatment on cell survival. MTT solution (5 mg/mL) was added to each well and kept at 37°C for 4 h. Subsequently, the medium containing MTT was discarded, and 200 *μ*L dimethyl sulfoxide (DMSO) was added to dissolve the formazan crystals. The absorption of the resulting solubilized formazan was measured in a plate reader at 570 nm, and cell viability was calculated as percent of control.

### 2.4. Enzyme-Linked Immunosorbent Assay (ELISA)

To investigate the effect of MPP on the inflammatory response of cancer cells, the levels of secreted cytokines, including TNF-*α*, IL-1*β*, and IL-6, were evaluated in LPS-stimulated breast and ovarian cancer cells with or without treatment with either MPP or the combination of MPP and curcumin. The levels of secreted cytokines were assessed in the cell culture supernatant by ELISA, using kits from Abcam, USA. All the kits were designed based on sandwich method using the colorimetric detection technique and were suitable for the measurement of cytokines in cell culture supernatant. Capture antibody, specific for the analyte, and the detector antibody were used in each assay, and the development of the colored compound was performed by TMB substrate. Recombinant TNF-*α*, IL-1*β*, and IL-6 were used for generating the standard curve to estimate the concentration of each cytokine. The sensitivity of the assay kits was 4.32 pg/mL, 5.64 pg/mL, and 1.6 pg/mL, for TNF-*α*, IL-1*β*, and IL-6, respectively.

### 2.5. NF-*κ*B Activity Assay

The effect of MPP alone or together with curcumin on the NF-*κ*B activity was inspected by analyzing the DNA-binding ability of NF-*κ*B in the nuclear extracts of the cells, by NF-*κ*B p65 Transcription Factor Assay Kit (Abcam, UK), using a double-stranded DNA sequence containing the NF-*κ*B response element. Briefly, cells were harvested, and the nuclear extracts were prepared using nuclear extraction buffer containing protease and phosphatase inhibitors and nonidet P-40 as the detergent. The protein levels were assessed by bicinchoninic acid (BCA) assay. The nuclear extracts were added to the wells in a 96-well plate holding the immobilized DNA comprising the NF-*κ*B response element. Detection was done by NF-*κ*B primary antibody, followed by HRP-conjugated goat anti-rabbit antibody. After incubating and washing, the absorbance of the developed color was measured at 450 nm, by means of a plate reader. The absorbance of each of the treatment groups was compared with that of the control group.

### 2.6. Real-Time PCR

The expression of genes of interest, including *COX2* encoding cyclooxygenase, *INOS* encoding inducible nitric oxide synthase, and *MMP9* encoding matrix metalloproteinase 9, was assayed using real-time PCR. RNA was isolated from cells, using RNeasy mini kit (QIAGEN, Germany), according to the manufacturer's instructions. The extracted RNA was reverse transcribed into cDNA using the first-strand cDNA synthesis kit (BioFact, Daejeon, South Korea), as stated by the manufacturer's protocol. Semiquantitative real-time PCR was conducted by SYBR green kit (Ampliqon, Denmark), using specific primers (Table 1). The PCR reaction was performed in three steps, with an initial denaturation step of 95°C for 10 minutes, followed by 40 cycles of 95°C for 15 seconds and 60°C for 60 seconds. *GAPDH* gene was used as the normalizer, and the analysis of the results was carried out by *ΔΔ*Ct method.

### 2.7. Griess Assay for the Measurement of Nitrite

Due to the short half-life of NO, its direct measurement is challenging. Therefore, the concentrations of nitrite and nitrate, which are stable end products of NO degradation, were measured to estimate NO production. The Nitrite Assay Kit, based on the Griess method, was purchased from Sigma-Aldrich (Germany) and was utilized for the estimation of nitrite levels. For this assay, 100 *μ*L of cell culture supernatant was prepared and added to 100 *μ*L of Griess reagent and kept at room temperature for 10 minutes. The absorption of the mixture was then measured by a microplate reader at 540 nm.

### 2.8. Docking Analysis

Docking was executed by AutoDock4 software using a semiflexible docking process to insert MPP and curcumin into the binding site of the stiff target. The study was based on a 2.3 Å resolution X-ray crystallographic structure of NF-*κ*B1 in complex with DNA (identifying code in RCSB: 1NFK). AutoDock tools from Molecular Graphics Laboratory (MGL) tool package (1.5.7 version) were used to prepare the target and ligands. Kollman partial charges and polar hydrogens were added after the cocrystallized macromolecule (DNA) and water molecules were extracted. In the docking simulations, we used p50 dimers and monomers (chains A and B). HyperChem 8.0 was used to minimize the ligands' energy utilizing the MM+ approach (molecular mechanics force field). A sequence of active site residues, engaged in hydrogen bonding with the NF-*κ*B recognition site of DNA, was used to define the enclosing box's coordinates (*x* = −1, 1958 angstroms, *y* = 9.0149 angstroms, and *z* = 19, 7598 angstroms). These amino acids include Arg54, Arg56, Tyr57, Cys59, Lys241, Gln306, and Thr143 [23].

### 2.9. Western Blotting

The cell lysate was prepared after treatment using RIPA buffer, and its total protein content was measured by the Bradford method. Equal amounts of proteins were loaded in SDS-PAGE, and the separated proteins were then blotted onto PVDF membranes. After overnight blocking with skim milk, the membranes were incubated with a 1 : 1000 dilution of the primary antibodies against I*κ*B*α* and *β*-actin (Sigma-Aldrich, Germany), for an additional overnight at 4°C. Horseradish peroxidase (HRP)- conjugated secondary antibody (Santa Cruz Biotechnology, UK) was applied to the membranes and incubated for 1 h at room temperature and in the dark. ECL substrate was used to develop bands, which were visualized on X-ray film.

### 2.10. Statistical Analysis

GraphPad Prism software version 6.07 (GraphPad Software, San Diego, CA) was used for data analysis. One-way analysis of variance (ANOVA) followed by Tukey's post hoc test was used to evaluate differences between the experimental groups. Data were presented as mean ± standard deviation (SD), and *P* < 0.05 was considered statistically significant.

## 3. Results

### 3.1. MPP and Its Combination with Curcumin Reduced Cell Viability

**Figure 2:**
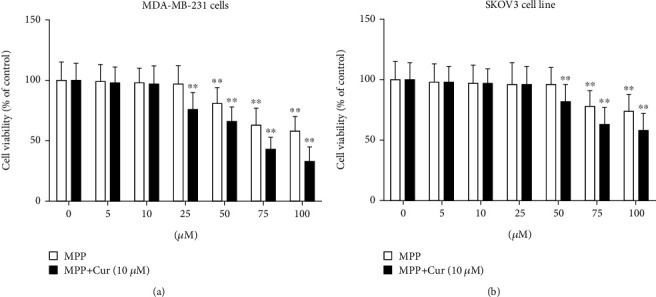
The viability of (a) MDA-MB-231 cells and (b) SKOV3 cells in response to various concentrations of MPP with or without 10 *μ*M curcumin assessed by MTT assay in 96-well plate format, repeated 3 times. ^∗∗^*P* < 0.01 compared with control.

MTT assay results showed that MPP could significantly decrease cell viability (Figure 2). The concentrations greater than 50 *μ*M and 75 *μ*M of MPP were effective in reducing the viability of MDA-MB-231 and SKOV3 cells, respectively. The addition of curcumin to the treatment medium enhanced the cytotoxic effect of MPP and decreased the lowest effective concentration to 25 *μ*M in MDA-MB-231 cells and 50 *μ*M in SKOV3 cells (Figure 2).

### 3.2. LPS-Induced NF-*κ*B DNA-Binding Activity Was Suppressed by MPP and Its Combination with Curcumin

**Figure 3:**
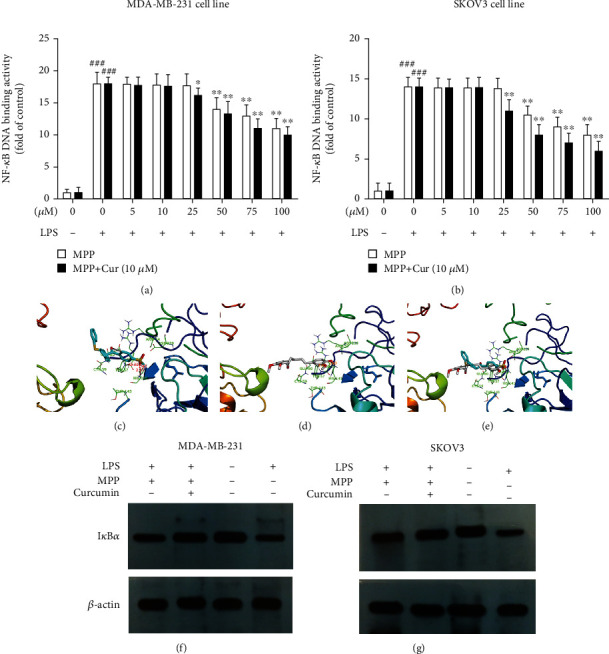
The DNA-binding activity of NF-*κ*B in LPS-stimulated (a) MDA-MB-231 cells and (b) SKOV3 cells, in response to various concentrations of MPP with or without 10 *μ*M curcumin. The error bar represents the standard deviation (SD) of mean from three separate experiments. ^###^*P* < 0.001 compared with untreated unstimulated control cells; ^∗^*P* < 0.05 and ^∗∗^*P* < 0.01 compared with LPS-stimulated untreated cells. The results of docking experiment, representing NF-*κ*B1 active site containing (c) MPP and (d) curcumin. (e) Superimposition of MPP (cyan) on curcumin (gray) in NF-*κ*B1 active site. The levels of I*κ*B in (f) MDA-MB-231 cells and (g) SKOV3 cells, normalized to *β*-actin levels, performed by Western blotting.

NF-*κ*B is a transcription factor that, upon activation, binds to specific sequences of DNA, inducing the expression of several genes in its signaling pathway. Therefore, to evaluate the therapeutic effect of MPP and curcumin on the NF-*κ*B pathway in MDA-MB-231 and SKOV3 cells, DNA-binding activity of this transcription factor was examined by an ELISA-based method. The results indicated that LPS significantly increased the DNA-binding activity of NF-*κ*B compared to the cells that received no LPS. Treatment with MPP, at concentrations of 50 *μ*M and higher, significantly reduced NF-*κ*B DNA-binding activity, compared to cells receiving LPS alone. Coadministration of curcumin increased the potency of MPP. When combined with curcumin, MPP reduced the activity of NF-*κ*B at 25 *μ*M concentration (Figures 3(a) and 3(b)).

The results of docking studies indicated the interaction of MPP and curcumin with NF-*κ*B. AutoDock4 and PyMOL (Open-Source) were used to visualize and analyze the results of the docking experiment. MPP and curcumin were docked to the active site of NF-*κ*B-p50 (NF-*κ*B1) to evaluate their probable mechanisms. Results showed that MPP and curcumin occupied the active site of NF-*κ*B1 containing Arg54, Tyr57, Arg56, Cys59, Thr143, Lys241, and Gln306 at their optimum position. MPP can form hydrogen bonds with tyrosine 57 (distance = 2.31 Å), His 141 (distance = 2.77 Å), and Glu60 (distance = 2.32 Å). With tyrosine 57 and His 141, curcumin could form hydrogen bonds (Figure 3). The distance between the atoms involved in the hydrogen bonding of the ligands with the amino acids was appropriate. Curcumin was superimposed over the other ligand (MPP), indicating that the two structures in the NF-*κ*B active site were in a similar position (Figures 3(c)–3(e)).

Considering I*κ*B as an essential regulator of NF-*κ*B activity, its protein level was evaluated in breast and ovarian cancer cells. As shown in Figure 3(f), LPS caused a remarkable decrease in the levels of I*κ*B in MDA-MB-231 cells, while MPP prevented this effect. The combination of curcumin and MPP was even more effective in the elevation of I*κ*B concentration. Same effect was observed in SKOV3 cells; MPP increased I*κ*B levels and the addition of curcumin enhanced this effect (Figure 3(g)).

### 3.3. MPP Diminished the Production of Cytokines, an Effect That Was Potentiated by Curcumin

**Figure 4:**
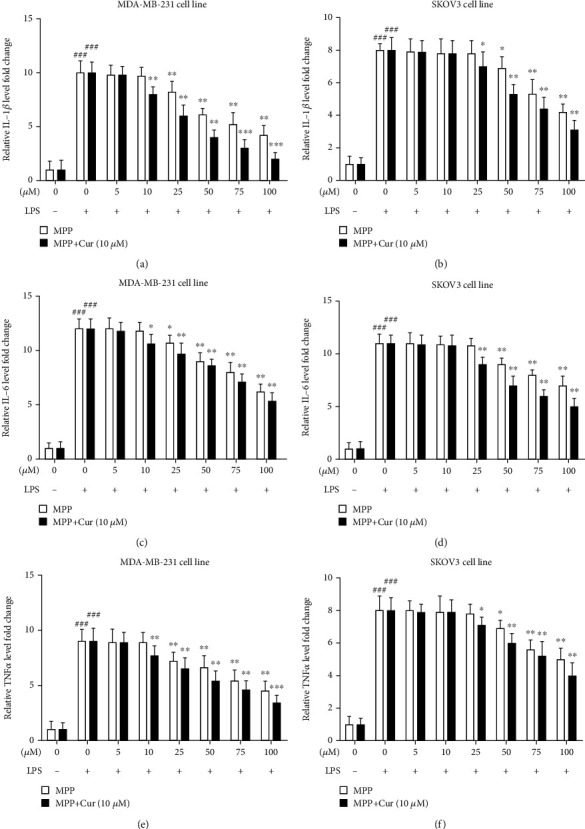
MDA-MB-231 breast cancer cell line and SKOV3 ovarian cancer cell line were treated with LPS to induce inflammation and subsequently were treated with different concentrations of MPP with or without curcumin. Subsequently, the levels of secreted cytokines were measured in the cell culture supernatant. The levels of IL-1*β* in (a) MDA-MB-231 and (b) SKOV3 cells; the levels of IL-6 in (c) MDA-MB-231 and (d) SKOV3 cells; and the levels of TNF-*α* in (e) MDA-MB-231 and (f) SKOV3 cells were measured. ^###^*P* < 0.001 compared with unstimulated untreated control cells; asterisks indicate comparison with LPS-stimulated untreated cells (^∗^*P* < 0.05, ^∗∗^*P* < 0.01, and ^∗∗∗^*P* < 0.001). The results are mean ± SD of at least three independent experiments.

Proinflammatory cytokines, including IL-1*β*, IL-6, and TNF-*α*, are produced in response to the stimulation of NF-*κ*B signaling pathway. The overproduction of cytokines exacerbates inflammation in the tumor microenvironment [15]. Thus, inhibiting NF-*κ*B activity and proinflammatory cytokines in the tumor microenvironment can suppress tumor development and growth by reducing inflammation. We evaluated the effect of MPP and curcumin on IL-1*β*, IL-6, and TNF-*α* production in MDA-Mb-231 and SKOV3. As shown in Figure 4, the addition of LPS significantly increased the relative levels of cytokines compared to the control group. MPP significantly attenuated the LPS-induced secretion of all measured cytokines in MDA-MB-231 cells and SKOV3 cells. The addition of curcumin to MPP potentiated this inhibitory effect and reduced the effective concentration of MPP (Figure 4).

**Figure 5:**
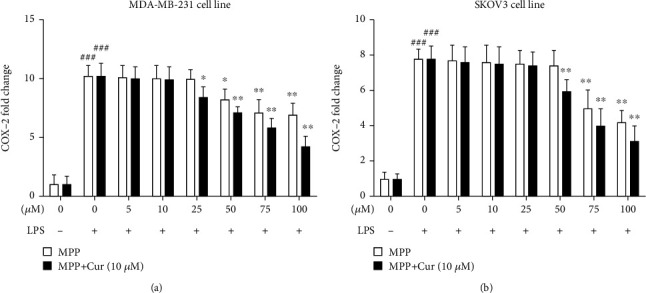
Gene expression of COX2 in LPS-stimulated (a) MDA-MB-231 and (b) SKOV3 cells, in response to various concentrations of MPP, either alone or combined with 10 *μ*M curcumin. ^###^*P* < 0.001 compared with untreated unstimulated control cells; ^∗^*P* < 0.05, ^∗∗^*P* < 0.01, and ^∗∗∗^*P* < 0.001 compared with LPS-stimulated untreated cells. The results are mean ± SD of at least three independent experiments.

We also evaluated the expression of *COX2* gene. COX2 is the crucial enzyme in the biosynthesis pathway of prostaglandins and can be induced by cytokines such as TNF-*α* [24]. As shown in Figure 5, LPS caused a dramatic surge in the expression of the *COX2* gene, while MPP, either alone or together with curcumin, could effectively suppress the gene expression of *COX2* (Figure 5).

### 3.4. MPP-Curcumin Combination Effectively Reduced NO Production in MDA-MB-231 and SKOV3 Cells

**Figure 6:**
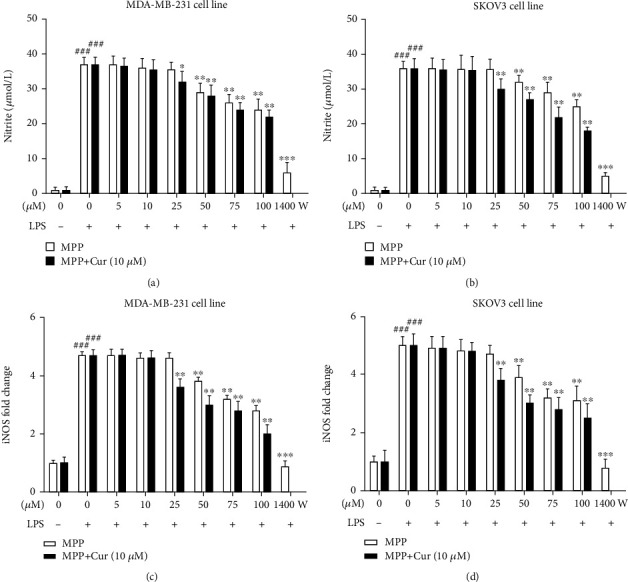
The levels of nitrite as the marker of NO production in LPS-stimulated (a) MDA-MB-231 and (b) SKOV3 cell lines. The gene expression of *INOS* in LPS-stimulated (c) MDA-MB-231 and (d) SKOV3 cell lines, measured by real-time PCR. ^###^*P* < 0.001 compared with untreated unstimulated control cells; asterisks indicate comparison with LPS-stimulated untreated cells (^∗^*P* < 0.05, ^∗∗^*P* < 0.01, and ^∗∗∗^*P* < 0.001); ^###^*P* < 0.001 compared with untreated unstimulated control cells. The experiment was repeated at least three times independently.

Nitrite is the major oxidative metabolite of NO [25], and so it can be evaluated for estimation of NO levels. LPS treatment resulted in a significant increase in nitrite levels (Figures 6(a) and 6(b)). Similarly, the expression of the *INOS* gene was also induced by LPS (Figures 6(c) and 6(d)). The expression of *INOS* gene, as well as nitrite production, was inhibited by MPP with the minimum concentration of 50 *μ*M in both cell lines. The addition of 10 *μ*M curcumin increased the inhibitory effect of MPP on *INOS* gene expression and nitrite production in MDA-MB-231 and SKOV3 cell lines (Figure 6).

### 3.5. MPP Downregulated MPP9 Expression, and Curcumin Boosted This Effect

**Figure 7:**
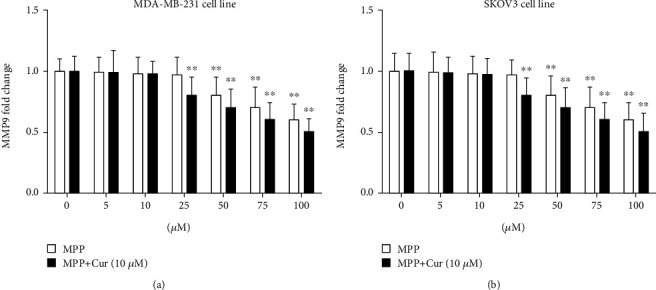
The expression of *MMP9* gene in (a) MDA-MB-231 and (b) SKOV3 cell lines, measured by real-time PCR. ^∗∗^*P* < 0.01 compared with untreated control cells. The results summarize the data of three separate experiments.

MMP9 is one of the essential extracellular matrix proteases, whose role in tumor metastasis processes is well known. The MPP9 gene is one of the downstream genes of NF-*κ*B signaling, whose expression is increased by this transcription factor [26]. In this study, the expression level of *MMP9* gene in MDA-MB-231 and SKOV3 cells exposed to MPP and MPP-curcumin combination was evaluated by real-time PCR. As shown in Figure 7, MPP at concentrations of 50 *μ*M and higher significantly reduced *MMP9* gene expression compared to control cells. Coadministration of 10 *μ*M curcumin further reduced the expression of the *MMP9* gene (Figure 7).

## 4. Discussion

Curcumin is a well-known natural product with numerous pharmacological effects, especially in cancer [27]. Part of the chalcone backbone is comparable to curcumin [28], and thus, it might be a suitable choice to be administered along the synthetic chalcones. It has been shown in recent studies that curcumin inhibits cell proliferation, promotes apoptosis, suppresses chemotherapeutic resistance, and exerts antimetastatic effects in the treatment of both breast and ovarian cancers [29–32], either alone or combined with other chemotherapeutic agents [33–35]. Here, we showed that a chalcone derivative could effectively suppress cell viability and adding curcumin significantly increased the potency of MPP on the viability of breast and ovarian cancer cells. Consistently, coadministration of curcumin with garcinol, a polyisoprenylated chalcone, showed a high synergism on proliferation and survival of pancreatic cancer cells [36].

Inflammatory pathways are involved in the survival, proliferation, angiogenesis, invasion, and metastasis of cancer. Chalcones have been reported to suppress NF-*κ*B-mediated inflammation, and subsequently the cancer progression [28]. Studies on NF-*κ*B pathway revealed that this signaling pathway is strongly involved in breast and ovarian cancer pathogenesis [37, 38]. Therefore, targeting NF-*κ*B and its downstream genes may be a novel therapeutic strategy for treating these cancers. We revealed in this study that MPP could exert an inhibitory effect on NF-*κ*B. It was able to attenuate the DNA-binding activity of NF-*κ*B, probably by directly interacting with its active site. On the other hand, MPP attenuated LPS-induced degradation of I*κ*B, an essential inhibitor of NF-*κ*B. MPP was even more effective in reverting the I*κ*B level to its average concentration when combined with curcumin. The reduction of the levels of NF-*κ*B downstream targets, including inflammatory cytokines and the expression of COX2, further confirmed the suppression of the NF-*κ*B pathway by MPP. In line with our findings, various chalcones have been shown to act as inhibitors of NF-*κ*B. For example, butein suppresses the NF-*κ*B activation induced by different inflammatory factors and carcinogens [39]. Shen et al. reported an inhibitory effect on NF-*κ*B for the basic structure of chalcone (1,3-diphenyl-2-propenone) [40]. Hydroxysafflor yellow A also inhibits NF-*κ*B nuclear translocation and its DNA-binding activity, as well as suppression of proinflammatory cytokines including TNF-*α*, IL-1*β*, and IL-6 [41].

Lin et al. showed that *in vitro* inhibition of NF-*κ*B by curcumin suppresses cell proliferation and angiogenesis in ovarian carcinoma [42]. In this study, we showed that curcumin increased the potency of MPP in the suppression of inflammatory cytokines in both breast and ovarian cancer cells. The presence of inflammatory mediators in the tumor microenvironment is a hallmark of breast and ovarian cancer. IL-1*β* blocking leads to improved antitumor cell immunity [43]. Similarly, inhibition of IL-6 can lead to the suppression of colony formation, reduction of cell survival, and repression of tumor growth in breast cancer [44]. Therefore, MPP alone or combined with curcumin might be considered an efficient tool to reduce inflammation by blockade of NF-*κ*B pathway and dampening the release of the inflammatory cytokines.

Increased expression of iNOS and overproduction of NO have been found in many types of cancers, especially breast and ovarian cancers [45, 46]. There is a strong correlation between high iNOS expression and aggressiveness and poor survival in patients with breast and ovarian cancers [46, 47]. Inhibitors of iNOS are able to reduce tumor initiation and invasion in triple-negative breast cancer [45]. Our results showed that our chalcone derivative could effectively suppress *INOS* gene expression and NO production in both breast and ovarian cancer cells, an effect enhanced by curcumin. Similar to our findings, anti-inflammatory activity has been reported for cardamonin in cellular models of inflammation, which is due to declined iNOS and COX2 expression and NO synthesis [48]. Some other chalcone compounds, including 4-hydroxylonchocarpin and 2′,5′-dihydroxy-4-chloro-dihydrochalcone, also prevent LPS-stimulated augmentation of iNOS expression [49, 50]. Suppression of iNOS activity and scavenging activity on NO has also been previously reported for curcumin in breast cancer models [51].

Another downstream target of NF-*κ*B is MMP9, which has a particular binding site for NF-*κ*B on its promoter [52] and is directly associated with the invasiveness of breast cancer [53]. Here, we showed that the gene expression of MMP9 was downregulated by MPP. Curcumin increased the efficacy of MPP in the downregulation of MMP9. Consistently, anti-invasive properties have been proposed for chalcones [54]. Some chalcones, such as isoliquiritigenin and butein, have been shown to decrease MMP9 and inhibit tumor cell invasion and metastasis in prostate and leukemia cells, respectively [39, 55]. The inhibitory effect of curcumin on TGF-*β*-induced expression of MMP9 has also been previously reported in MDA-MB-231 breast cancer cells, so it is plausible that the inclusion of curcumin into the treatment media would enhance the suppressive effect of MPP on *MMP9* gene expression.

Molecular mechanisms and signal transduction pathways that affect carcinogenesis are numerous and complicated; thus, other signaling mechanisms might also be responsible for the effect of MPP and its combination with curcumin on the cancer cells. Here, we focused on the NF-*κ*B pathway, due to its importance in breast and ovarian cancer cells; however, off-target effects might also be possible, which requires further studies.

## 5. Conclusion

In conclusion, our findings suggest that MPP, a novel synthetic derivative of chalcone, alone or in combination with curcumin, could serve as an effective anti-inflammatory and cytotoxic treatment for combating breast and ovarian cancer, mediated by the inhibitory effect of this combination on NF-*κ*B activity and its downstream genes. However, further pharmacokinetic and animal studies are required to establish the value of the presented substances as adjuvant therapy for breast and ovarian cancer.

## Figures and Tables

**Table 1:** Specific primers used in real-time PCR analysis.

Gene	Forward (5′–3′)	Reverse (5′–3′)
COX2	TTCAAATGAGATTTGGGAAAAT	AGATCATCTGCCTGAGTATCTT
iNOS	GTTCTCAAGGCACAGGTCTC	GCAGGTCACTTATGTCACTTATC
MMP9	TGTACCGCTATGGTTACACTCG	GGCAGGGACAGTTGCTTCT
GAPDH	ACCCACTCCTCCACCTTTGA	CTGTTGCTGTAGCCAAATTCGT

## Data Availability

Data will be made available upon reasonable request from corresponding author.
